# DNMT3A binds ubiquitinated histones to regulate bivalent genes

**DOI:** 10.1038/s41588-022-01073-4

**Published:** 2022-05-01

**Authors:** Aled J. Parry, Wolf Reik

**Affiliations:** 1 Epigenetics Programme, The Babraham Institute, Cambridge, UK, CB22 3AT; 2 Altos Labs Cambridge Institute, Cambridge, UK, CB21 6GP

## Abstract

A new study demonstrates that the disordered N-terminal domain of DNMT3A1 binds PRC1-catalyzed H2AK119ub, targeting DNA methylation to bivalent promoters in mouse brain cortical cells. Methylation around bivalent genes is critical for mouse postnatal development, and could be equally important in other cell types and in disease.

DNA methylation (largely 5-methylcytosine in mammals, 5mC) is an important epigenetic modification associated with gene expression levels during development, in aging and in disease ^1^. It is added *de novo* by DNA methyltransferases 3A, 3B and 3C (DNMT3A/B/C) and maintained across cell divisions by DNMT1^1^. *Dnmt3a* is essential for postnatal development: knockout mice are runted and die at ~4 weeks of age^2^. In humans, *DNMT3A* mutations are associated with paragangliomas^3^, microcephalic dwarfism^4^ and cancer^5^. There are two distinct isoforms of DNMT3A: DNMT3A1 and DNMT3A2 (Fig. 1). Both contain PWWP and ADD domains that bind H3K36me3 (trimethylation of lysine 36 on histone H3) and unmodified H3K4, respectively^6,7^. DNMT3A1, however, contains a predicted N-terminal disordered domain – it does not have significant secondary structure according to computational models based on amino acid composition – which is lacking from DNMT3A2 (Fig. 1). Little is currently known about the respective functions of these isoforms or about their individual contributions to development and disease. In this issue of *Nature Genetics*, Gu, Hao et al.^8^ show that the disordered N-terminal domain of DNMT3A1 is essential for neuronal gene expression and mouse postnatal development.

To understand the difference between the two isoforms of *Dnmt3a* the authors generated isoform-specific knockout mice and found that, whilst *Dnmt3a2*^-/-^ mice had no altered phenotype, *Dnmt3a1*^-/-^ mice were similar to *Dnmt3a*^-/-^ knockouts^2,8^. Because they found that *Dnmt3a1* is predominantly expressed in the brain, and indeed re-expression in the brain could partially rescue the knockout phenotype, they focused here for mechanistic experiments.

Isoform-specific chromatin immunoprecipitation and sequencing (ChIP-seq) of DNMT3A1 in the cerebral cortex revealed enrichment flanking bivalent neurodevelopmental gene promoters, which was dependent on the disordered N-terminal domain (as ChIP-seq of N-terminal deletion mutants lost this enrichment). Bivalency is the co-occurrence of histone modifications associated with gene activation (H3K4me3) and repression (H3K27me3 and H2AK119ub) across the same region. It is characteristic of promoters regulating developmental genes that are not expressed at high levels but are poised for activation upon stimulation; for example, following activation of a signaling pathway that induces differentiation^9,10^. Thus, the authors show that DNMT3A1 flanks the promoters of genes that are important for neuronal development. The authors also detected DNMT3A1 binding at enhancer elements, but we do not yet know what role it plays here. Enhancers and bivalent domains are enriched for 5-hydroxymethylcytosine (5hmC)^11,12^, generated through oxidation of 5mC. 5hmC also accumulates in neurons, more so than in many other somatic cell types^13^, so perhaps DNMT3A1 also plays a role in the accumulation of this poorly understood epigenetic modification.

DNMT3A1 depletion resulted in hypomethylation around bound genes in cells from the cerebral cortex of knockout mice, and RNA sequencing (RNA-seq) experiments largely revealed downregulation of bivalent neurodevelopmental genes relative to wild type controls^8^. Contrarily, RNA-seq of sorted neuronal nuclei revealed both up- and down-regulation of genes in response to *Dnmt3a1* knockout, and only a weak correlation between hypomethylation and differential gene expression. The different transcriptional changes in the cortex (a heterogeneous tissue) and sorted neuronal cells is intriguing and warrants further investigation – does Dnmt3a1 function differently in glia? Promoter methylation is traditionally thought to be gene repressive, but methylation at the flanks of bivalent domains could also activate gene expression if it is important to constrain polycomb activity and thus the distribution of repressive histone modifications^14^. Loss of DNMT3A1 would also presumably result in loss of 5hmC, which could also have a regulatory role.

Next, the authors asked how the N-terminal domain of DNMT3A1 confers binding to bivalent domains. Computational analysis identified a putative ubiquitin binding region (UBR) within a predicted alpha-helix at amino acids 189-206. *In vitro* nucleosome pulldown assays confirmed that the N-terminal domain interacts with H2AK119ub, and this interaction was abrogated upon mutation of the UBR. A similar domain was described by Weinberg *et al.*^15^ who demonstrated that, in mesenchymal stem-like cells (MSCs), DNMT3A1 is capable of binding H2AK119ub at bivalent genes via the N-terminal disordered region. Intriguingly they only observed this when the PWWP domain of DNMT3A was mutated, as it is in some diseases^3–5^. Moreover, they show that this binding is dependent on H2AK119ub, catalyzed by polycomb repressive complex 1 (PRC1), but independent of PRC2 and H3K27me3^15^.

Together these works suggest that DNMT3A1 can be targeted by different molecular mechanisms in different contexts. Likely in some cell types targeting through the PWWP and ADD domains is dominant. In other contexts, including in neurons and in certain diseases, binding via the N-terminal domain is more common (Fig. 1). It will be important to determine what regulates DNMT3A1 targeting – perhaps post-translational modifications (PTMs), different enrichment of the epigenetic marks that direct DNMT3A1 and/or the relative abundance of co-factors. Mutational hotspots in the PWWP domain that increase H2AK119ub binding in MSCs^15^ are certainly a good place to begin looking for regulatory PTMs. Differential isoform usage is another route by which cells could govern DNMT3A activity – DNMT3A2 is the dominant isoform in embryonic stem cells and Gu, Hao *et al.* show that it is also expressed in bone marrow, spleen, testis and thymus. DNMT3A1 is the predominant isoform in the brain, kidneys and liver^8^. Perhaps, then, targeting of DNMT3A to bivalent domains is not desirable in certain tissues, such as pluripotent cells, and it would be interesting to understand whether the lack of this binding activity is important for transcriptional programs and/or pluripotency. Indeed, aberrant binding of DNMT3A to H2AK119ub in contexts where the PWWP domain is mutated, such as in paragangliomas and microcephalic dwarfism^3,4^, could be a mechanism that drives pathologies.

Overall, the study by Gu, Hao *et al.*^8^ in this issue together with the recent publication by Weinberg *et al.*^15^ identify an exciting molecular cross talk between PRC1, H2AK119ub and DNMT3A1 that is dependent on a novel UBR in the N-terminal disordered domain. This isoform, and specifically the N-terminal domain, is critical for neurodevelopmental gene expression and mouse postnatal development. Targeting of DNMT3A1 in different cell types is an interesting area of future research that may be of (patho)physiological importance.

## Figures and Tables

**Fig. 1:**
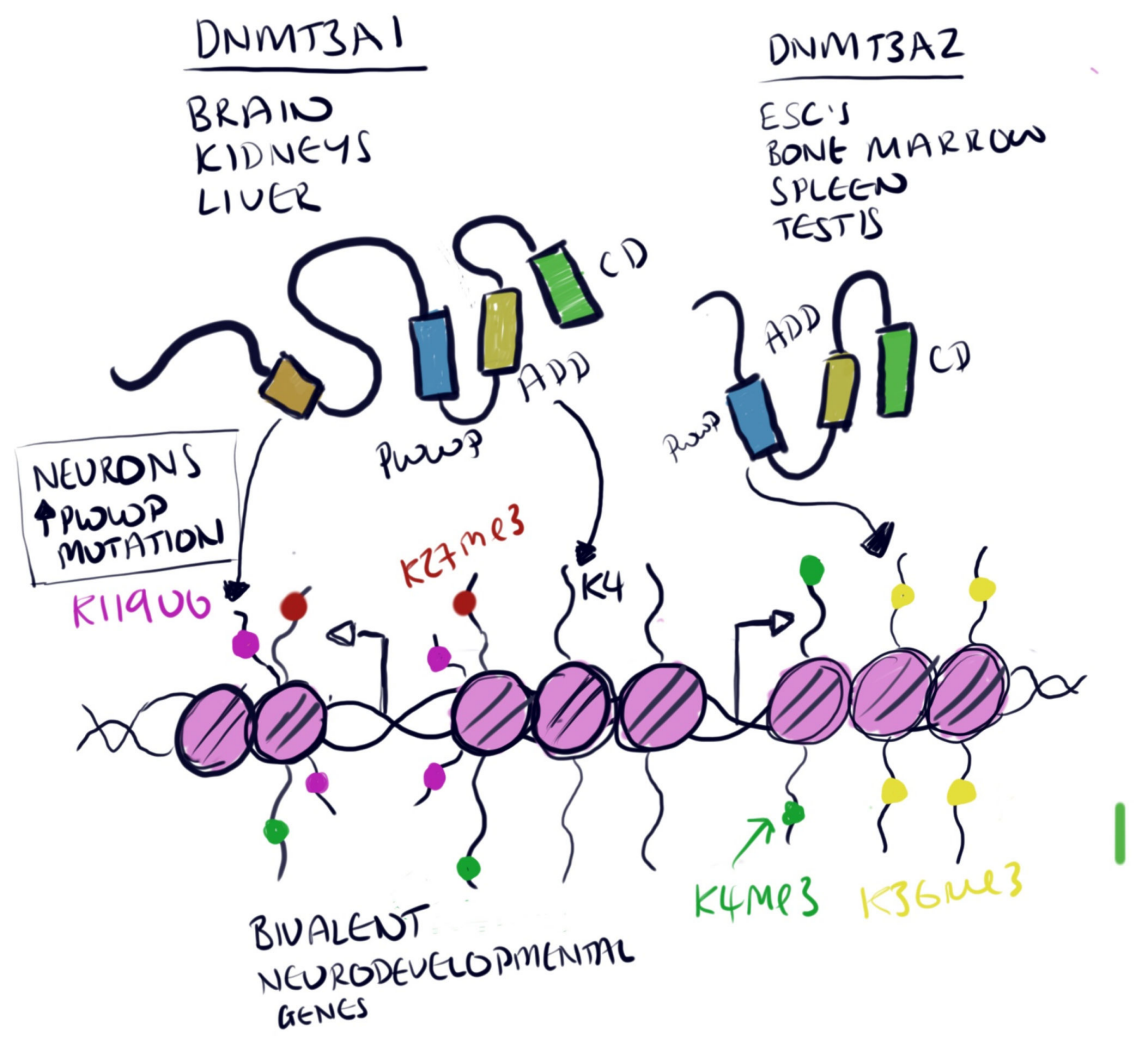
DNMT3A1 has a disordered N-terminal domain, missing from the other isoform DNMT3A2, that binds ubiquitinated lysine 119 on histone H2A (H2AK119ub). This binding directs DNMT3A1 to bivalent domains (H3K4me3^+^/H3K27me3^+^) in some contexts such as in neurons or where the PWWP domain is mutated.
